# Neoadjuvant Advantage, Adjuvant Challenge: Clinical Evidence and Hypothetical Biological Mechanisms Behind Divergent Tolerability

**DOI:** 10.3390/cancers18193137

**Published:** 2026-09-28

**Authors:** Patrícia Dias, Joaquim Faria Monteiro, Maria João Rocha, Daniel Moreira-Gonçalves, Lúcio Lara Santos, Jorge Gonçalves, Paula Fresco

**Affiliations:** 1 Laboratory of Pharmacology, Department of Drug Sciences, Faculty of Pharmacy, University of Porto, 4050-313 Porto, Portugal; padias@ff.up.pt (P.D.); j.monteiro@ff.up.pt (J.F.M.); mjrocha@ff.up.pt (M.J.R.); pfresco@ff.up.pt (P.F.); 2 Mechanistic Pharmacology and Pharmacotherapy, UCIBIO Applied Molecular Biosciences Unit, Associate Laboratory i4HB, Institute for Health and Bioeconomy, Faculty of Pharmacy, University of Porto, 4050-313 Porto, Portugal; 3 Research Center in Physical Activity, Health and Leisure (CIAFEL), Faculty of Sports, University of Porto (FADEUP), 4200-450 Porto, Portugal; 4 Laboratory for Integrative and Translational Research in Population Health (ITR), 4050-600 Porto, Portugal; 5 Experimental Pathology and Therapeutics Group, Research Centre of IPO Porto and RISE@CI-IPOP (Health Research Network), 4200-072 Porto, Portugal; lucio.santos@ipoporto.min-saude.pt; 6 Department of Surgical Oncology, Portuguese Oncology Institute of Porto, Porto Comprehensive Cancer Centre, 4200-072 Porto, Portugal

**Keywords:** adjuvant chemotherapy, neoadjuvant therapy, gastrointestinal neoplasms, breast cancer, pharmacokinetics, perioperative oncology

## Abstract

Adjuvant chemotherapy is a cornerstone of curative treatment for solid tumours; however, its benefit is frequently limited by poor tolerability and low treatment completion rates after surgery. Importantly, tolerability decreases from neoadjuvant to adjuvant regimens, a phenomenon particularly evident in gastrointestinal malignancies and observed in breast cancer, despite the latter generally involving less extensive surgery. Moreover, the mechanisms underlying this reduced tolerability remain a conundrum in clinical practice. This narrative review explores the discrepancy between neoadjuvant and adjuvant chemotherapy tolerability in gastrointestinal and breast cancers, focusing on potential biological and pharmacological determinants. Clinical and translational evidence is analysed, including treatment completion, dose intensity, and toxicity profiles in perioperative settings (i.e., any chemotherapy around the time of surgery, e.g., preoperative, intraoperative, and immediately postoperative chemotherapy), with emphasis on conventional cytotoxic regimens. Across tumour types, neoadjuvant chemotherapy is consistently associated with higher completion rates and greater dose intensity than adjuvant treatments. Although postoperative morbidity, nutritional impairment, sarcopenia, organ dysfunction and delayed recovery contribute to reduced tolerance and may prevent the initiation or completion of postoperative chemotherapy, particularly in gastrointestinal cancers, these factors may not fully account for the observed differences across treatment settings. We therefore discuss additional pharmacokinetic and tumour-related hypotheses as complementary explanations requiring clinical validation. Emerging evidence indicates that tumour burden and surgical resection may modify systemic drug handling, including pharmacokinetics and tissue distribution. The removal of a tumour could decrease the sequestration of drugs and change the interactions between the host and the drugs, which might narrow the therapeutic index after surgery. Consequently, the tolerability of adjuvant chemotherapy could be linked to both postoperative vulnerability and potential biological changes resulting from tumour removal. Optimising treatment sequencing will require integration of pharmacological, physiological, and tumour-specific factors to develop personalised strategies that balance treatment efficacy and quality of life.

## Simple Summary

This review explores why chemotherapy given after surgery is often harder to complete and less well tolerated than treatment given before surgery, especially in gastrointestinal and breast cancers. It aims to compare these treatment settings and to understand the biological and drug-related reasons behind this difference. By clarifying these mechanisms, the review may help researchers and clinicians design better treatment schedules, improve tolerability, and develop more personalised strategies that maintain effectiveness while reducing harm to patients.

## 1. Introduction

Adjuvant chemotherapy of solid tumours is limited by the inability of a substantial proportion of patients to initiate or complete postoperative chemotherapy treatment, despite unequivocal survival benefit. This limitation is particularly evident in gastrointestinal malignancies. In contrast, identical or closely related regimens administered in the neoadjuvant setting: (a) are reproducibly better tolerated as they produce fewer burdensome effects, which helps patients maintain compliance and quality of life; (b) achieve higher completion rates, i.e., a successful delivery of the final prescribed cycle of the systemic chemotherapy; and (c) are characterised by a greater dose intensity; i.e., a higher amount of drug is delivered per unit of time. This divergence exposes a fundamental limitation in current treatment paradigms: systemic therapy is preferentially delivered at a time when host resilience is maximally compromised.

Divergent tolerance to neoadjuvant versus adjuvant chemotherapy in gastrointestinal malignancies is often attributed to the impact of major surgical procedures, such as gastrectomy, hepatectomy or rectal resection, which induce profound physiological disruption, including catabolism, systemic inflammation and organ dysfunction, thereby increasing postoperative morbidity and nutritional impairment and reducing the patient’s capacity to tolerate chemotherapy. However, these surgery-related factors do not fully account for the consistency and magnitude of the tolerability gap observed across different gastrointestinal tumour types, nor for reports of higher adverse event rates with adjuvant compared with neoadjuvant chemotherapy in breast cancer [1], a disease in which the surgical procedure is not expected to cause marked nutritional compromise or major metabolic disruption.

The persistence of this pattern in breast cancer strongly suggests that additional biological determinants are operative beyond surgical burden alone, possibly at the level of tumour–host interactions, given that removal of the primary tumour mass is the common denominator in all these settings. This possibility challenges the conventional view of tumours as mere passive therapeutic targets and instead emphasises their role as active modulators of systemic drug handling.

The review will first synthesise data on completion rates, toxicity profiles (i.e., the specific range, severity, and timeline of adverse side effects caused by chemotherapeutic drugs), and survival outcomes, integrating clinical evidence gathered from gastrointestinal and breast malignancies to present the available evidence that supports that adjuvant chemotherapy presents inferior tolerability and completion rates compared with neoadjuvant chemotherapy. It further examines mechanistic hypotheses, including postoperative pharmacokinetic perturbations, redistribution phenomena following tumour debulking, and tumour-mediated modulation of drug exposure. Finally, the review addresses emerging strategies to overcome reduced chemotherapy tolerability when moving from neoadjuvant to adjuvant chemotherapy. We also highlight the need for innovative trial designs explicitly aimed at optimising treatment sequencing, with the goal of aligning therapeutic efficacy with tolerability and long-term patient outcomes.

## 2. Methods

We used PubMed as the bibliographic database and selected eligible publications from 1983 to 2026. Our primary focus was on clinical evidence related to the reduced tolerability and lower treatment compliance observed in adjuvant regimens compared to neoadjuvant regimens. To address this question, the following keywords were added to the query box: (neoadjuvant AND adjuvant) and (FLOT chemotherapy), (FOLFOX OR ECF OR fluoropyrimidines OR taxanes OR platinum compounds) and (adjuvant chemotherapy AND solid tumours), (gastric cancer AND neoadjuvant AND adjuvant), (colorectal cancer AND neoadjuvant AND adjuvant), (breast cancer AND neoadjuvant AND adjuvant). Next, we explored potential factors and underlying mechanisms that could explain the differences in tolerability between neoadjuvant and adjuvant chemotherapy. We added the following keywords to the query box: (tolerability AND mechanisms AND adjuvant chemotherapy) and (surgery-related factors AND adjuvant chemotherapy). Finally, we investigated the literature related to our central mechanistic proposition, i.e., the “tumour-sink effect”. We added the following keywords to the query box: (chemotherapy AND tumour sink-effect) and (chemotherapy AND tumour AND sequestration). Our eligibility criteria prioritised clinical, pharmacokinetic and mechanistic evidence and included only peer-reviewed journal articles or book chapters published in English. The search had no exclusion criteria.

## 3. Clinical Evidence

Clinical studies indicate that adjuvant chemotherapy generally exhibits inferior tolerability and lower completion rates when compared to neoadjuvant chemotherapy. However, it is important to note that the clinical evidence varies significantly across different cancer types, including gastric, colorectal, and breast cancer. Several factors influence this variability, including disease stage, chemotherapy regimens, the incorporation of radiotherapy, timing of surgical interventions, patient selection criteria, treatment objectives, endpoint definitions, and prevailing treatment standards. Key clinical studies comparing neoadjuvant and adjuvant chemotherapy are summarised in Table 1, which reports treatment delivery, toxicity, and outcomes across tumour types and regimens.

### 3.1. Gastric Cancer

The FLOT regimen, which consists of combined administration of 5-fluorouracil (5-FU), leucovorin, oxaliplatin, and docetaxel, is widely accepted as the standard for perioperative treatment of locally advanced gastric cancer. Perioperative evidence consistently shows that neoadjuvant FLOT-based chemotherapy is well tolerated and highly deliverable. However, poor tolerability often compromises the adjuvant phase, leading to substantial postoperative attrition and reduced delivery. In a retrospective cohort of 171 patients treated with four cycles of neoadjuvant FLOT followed by surgery, only 105 of 171 patients (61.4%) completed all four planned FLOT cycles. Among the 66 patients who did not complete all four planned postoperative cycles, 37 required de-escalation to FOLFOX (FLOT without docetaxel) because of poor Eastern Cooperative Oncology Group (ECOG) performance status, impaired oral intake, hematologic and neurologic toxicity, or cardiovascular events, and 29 received no postoperative chemotherapy, mainly due to postoperative deterioration and complications [2]. Disease-free and overall survival were significantly worse in patients who received no adjuvant therapy, underscoring that poor tolerability and consequent omission of postoperative chemotherapy are associated with worse prognosis [2].

A propensity-matched comparison of standard perioperative FLOT 4 + 4 (FLOT chemotherapy schedule with 4 neoadjuvant and 4 adjuvant cycles) versus total neoadjuvant FLOT x8 (no adjuvant; patients received all systemic chemotherapy before surgery) in resectable locally advanced gastric cancer further highlights the postoperative vulnerability of the adjuvant setting. The all-cycle completion rate was 89.1% (33/37 patients) in the total neoadjuvant group versus 67.6% (25/37 patients) in the FLOT 4 + 4 group, with 19% of patients (7/37 patients) in the FLOT 4 + 4 arm being unable to start any postoperative chemotherapy due to postoperative complications or intolerance, despite comparable complications occurring around the time of surgery between the two arms. Early survival outcomes (disease-free and overall survival) were similar, while pathological complete response rates tended to be higher with FLOT x8, suggesting that moving all cycles to the preoperative period can increase cumulative dose delivery without adding postoperative risk [3].

Real-world data from patients who received modified FLOT (the same components of FLOT in lower doses) also confirm a systematic drop in tolerability after surgery. In a cohort of 343 patients with resectable gastric cancer, 298 patients completed all four neoadjuvant cycles, whereas only 264 patients (77% of the entire cohort) completed the planned adjuvant chemotherapy. Grade 3–4 diarrhoea (42 patients), febrile neutropenia (16 patients), and postoperative frailty led to dose reductions, regimen changes, and treatment discontinuations [4]. In a phase II study of perioperative nab-paclitaxel plus FOLFOX (FOXAGAST), all six neoadjuvant cycles were generally feasible, but only half of operated patients resumed the same regimen postoperatively and nearly one third received no adjuvant chemotherapy, largely due to tolerance issues and performance status decline, despite high R0 resection rates and meaningful pathological regression [5].

The pivotal perioperative trials MAGIC and FLOT4 AIO that showed clear survival benefits for combined chemotherapy and surgery in resectable gastric and gastroesophageal junction cancer also exposed major limitations in the deliverability of postoperative treatment. In the MAGIC trial, perioperative ECF (epirubicin, cisplatin, and 5-FU) was associated with an increase in 5-year overall survival from 23% to 36% (HR 0.75). However, only approximately 55% of patients (137/250 patients) were able to initiate postoperative (adjuvant) chemotherapy, and only 40–42% completed all six planned cycles, underscoring that the adjuvant component had to be frequently omitted or curtailed despite the protocol design [6]. In FLOT4 AIO, perioperative FLOT extended median overall survival from 35 to 50 months and increased 3-year survival from 48% to 57% compared with ECF/ECX (viz., epirubicin, cisplatin, capecitabine). In this study, around 90% of patients (320/356 patients) completed all four preoperative cycles but only about 46% (162/356 patients) managed to complete all four postoperative cycles in the FLOT arm [7]. Together, these trials confirm that combined neoadjuvant and adjuvant chemotherapy is a standard of care but also consistently show that the adjuvant (postoperative phase) is less tolerable and is the most vulnerable part of the treatment pathway, with substantial loss of planned dose intensity occurring after major surgery even in highly selected clinical trial populations [6,7].

Collectively, these studies in patients with gastric cancer consistently show that adjuvant chemotherapy is less tolerable and more frequently omitted or modified, and that failure to deliver adjuvant therapy is associated with worse outcomes, requiring de-escalated postoperative regimens as an attempt to preserve survival in patients unable to tolerate adjuvant therapy.

### 3.2. Colorectal Cancer

In colorectal cancer (CRC), neoadjuvant and adjuvant systemic treatments are increasingly integrated into multimodality strategies, albeit with distinct patterns of use compared with gastric cancer. Consequently, the comparisons are influenced by multimodality design and treatment sequencing. In colon cancer, chemotherapy is still predominantly delivered in the adjuvant setting, after curative resection, with neoadjuvant therapy reserved for selected locally advanced cases. By contrast, in rectal cancer, neoadjuvant chemoradiotherapy, often within total neoadjuvant therapy frameworks that combine preoperative chemotherapy and radiation, plays a central role before surgery, with adjuvant chemotherapy used selectively. These differing configurations of pre- and postoperative systemic treatment provide an opportunity to examine how timing, cumulative exposure, and postoperative physiological vulnerability modulate toxicity when similar cytotoxic backbones are administered across the neoadjuvant and adjuvant phases.

In a phase II randomised trial involving 108 patients with locally advanced rectal cancer, participants were divided into two groups: arm A (52 patients) and arm B (56 patients). Patients in arm A received preoperative chemoradiation followed by surgery and four cycles of adjuvant capecitabine and oxaliplatin (CAPOX) [9,10]. In contrast, patients in arm B were given four cycles of CAPOX preoperatively, followed by chemoradiation and surgery. The postoperative adjuvant arm demonstrated a significantly higher incidence of adverse events compared to the induction arm, with rates of 17% versus 2%, respectively. Furthermore, grade 3 to 4 toxicity occurred in 54% of patients during the adjuvant phase, compared with 19% during the induction phase. The most common side effect was grade 3 diarrhoea, affecting 16% of patients in the adjuvant group versus 4% in the induction group. Treatment compliance was notably lower in the adjuvant arm (75%; 28 of 37 patients) than in the neoadjuvant regimen (96%; 52 of 54 patients) [9,10].

In addition, studies focused solely on adjuvant regimens in CRC may yield important insights into toxicity and prognosis in the postoperative setting.

In stage II–III CRC, evidence derives from large adjuvant case series and focuses on the relationship between chemotherapy toxicities, treatment modifications, and survival outcomes in the postoperative setting. A pooled analysis of 4,046 patients treated with various fluoropyrimidine- and oxaliplatin-based adjuvant regimens showed that most patients experienced some degree of toxicity within the first six months, with frequent dose reductions, treatment suspensions and early discontinuations, especially among those with higher-grade adverse events (3–4). When total adverse events were categorised by maximum CTCAE grade (versions 3.0 or 4.0) within six months, only 18% of patients had no reported toxicity (grade 0), while 24%, 37%, 19% and 1.4% experienced grades 1, 2, 3 and 4, respectively, reflecting how uncommon completely “toxicity-free” adjuvant treatment is in practice [8]. Importantly, the severity of these adverse events was itself prognostic compared to patients who had no toxicity signs; those with mild to moderate events (grades 1–3) had significantly better overall survival and relapse-free survival, whereas patients with very severe toxicity events (grade 4) had outcomes similar to, or worse than, the grade 0 group. Multivariable analyses confirmed that grade 1–3 total adverse events were associated with a lower risk of death and relapse, while grade 4 events did not confer benefits. These findings suggest that some toxicity may reflect adequate systemic exposure to chemotherapy, but excessive toxicity compromises treatment and survival. Similar patterns were observed when specific categories such as hematologic or skin toxicity were analysed, with moderate events correlating with improved prognosis [8].

Evidence from CRC studies conducted entirely in the adjuvant setting indicates considerable variability in postoperative treatment delivery. Dose reductions, treatment delays, discontinuations, and grade 3–4 adverse events provide distinct measures and should not be collapsed into a single construct of “tolerability”. Both insufficient and excessive toxicity are associated with poorer outcomes, whereas the most favourable prognoses are observed in patients who experience manageable, non-limiting adverse events throughout the adjuvant course.

### 3.3. Breast Cancer

In breast cancer, both neoadjuvant and adjuvant approaches are well-established, with neoadjuvant therapy increasingly used for locally advanced disease and to facilitate breast-conserving surgery. Accumulating randomised evidence indicates that neoadjuvant chemotherapy is at least as tolerable as the same systemic treatment delivered in the adjuvant setting and may be associated with fewer clinically relevant adverse events in women with operable breast cancer.

A meta-analysis of 14 randomised controlled trials, enrolling 5,500 women with operable breast cancer, found that neoadjuvant chemotherapy was associated with fewer adverse effects compared with the same regimens administered postoperatively, while overall survival was equivalent in both groups [1]. These findings were echoed in the Cochrane review, published by van der Hage and collaborators, which also reported fewer adverse effects with preoperative neoadjuvant chemotherapy, including a significantly lower risk of serious infection and a reduced likelihood of cardiotoxicity [12].

The European Cooperative Trial in Operable Breast Cancer (ECTO)—the only phase III trial to directly randomise patients to the same doxorubicin–paclitaxel—CMF regimen given either as adjuvant or as primary systemic (neoadjuvant) therapy in 1355 women—confirmed the feasibility and tolerability of this combination in both settings, with grade 3–4 toxicity rates below 5% across all arms [11]. Taken together, these data support the conclusion that neoadjuvant delivery of anthracycline-taxane regimens is at minimum as well tolerated as adjuvant administration and, in the context of the broader meta-analytic evidence, is associated with a more favourable adverse-effect profile. This difference is clinically meaningful, as failure to complete the planned adjuvant course has been independently associated with inferior outcomes, particularly in hormone receptor-negative and HER2-positive disease [1,12]. However, it must be considered that treatment completion is influenced by toxicity as well as postoperative complications, functional status, recurrence, patient preference, and clinician decision-making.

Over recent years, treatment paradigms have changed significantly. Indeed, recent evidence supports an increasingly individualised, subtype-specific approach to systemic therapy in breast cancer. In particular, treatment sequencing is determined not only by stage but also by tumour biology, with the option to use treatment response to guide postoperative escalation. In triple-negative breast cancer (TNBC), neoadjuvant chemotherapy has become particularly important because pathological complete response (pCR) provides important prognostic information, whereas residual disease identifies patients at increased risk of recurrence who may benefit from additional post-neoadjuvant therapy.

A meta-analysis of current evidence on survival outcomes with neoadjuvant versus adjuvant chemotherapy in TNBC showed substantially better survival among patients achieving pCR, while outcomes were poorer among those with residual disease after neoadjuvant chemotherapy [13]. More recent population-based studies suggest that, particularly in lower-risk T1-2N0 TNBC, neoadjuvant and adjuvant chemotherapy may achieve comparable survival, emphasising that treatment sequencing should be individualised rather than universally applied [14,15,16]. Contemporary management of higher-risk TNBC increasingly incorporates platinum-based chemotherapy and immune checkpoint inhibition, with response after neoadjuvant treatment informing postoperative strategies; patients with residual disease may receive escalation with capecitabine and, in appropriately selected patients with germline BRCA1/2 pathogenic variants, enzyme poly(ADP-ribose) polymerase (PARP) inhibition with olaparib. HER2-positive disease similarly illustrates the transition toward biologically tailored treatment, with neoadjuvant chemotherapy combined with HER2-targeted therapy enabling response-adapted postoperative management: continuation of HER2-directed therapy is appropriate after pCR, whereas residual invasive disease supports escalation to antibody-drug conjugate therapy. Collectively, these developments highlight a modern treatment paradigm in which neoadjuvant systemic therapy serves not only to downstage the tumour but also as an in vivo test of treatment sensitivity that guides post-neoadjuvant therapy.

Recent studies across TNBC, HER2-negative hormone receptor-positive disease, and invasive lobular carcinoma further underscore that the relative benefit of neoadjuvant versus adjuvant chemotherapy varies according to biological subtype and clinical risk, reinforcing the need for individualised treatment strategies [16,17,18,19]. Notably, tolerability profiles differ meaningfully across chemotherapeutics, HER2-targeted agents, immunotherapy, antibody-drug conjugates, and PARP inhibitors in breast cancer, with each class having distinct toxicities. Chemotherapy in breast-cancer regimens is mainly distinguished by haematological toxicity, while immunotherapy adds a different immune-mediated spectrum [20]. In early breast cancer, adding immune checkpoint inhibitors (ICIs) to standard therapy does not significantly raise overall all-grade or grade 3 adverse event risk, but it clearly increases endocrine immune-related adverse events such as hypothyroidism, hyperthyroidism, and adrenal insufficiency [21]. With HER2 monoclonal antibodies, cardiotoxicity is the main concern, with decreased left ventricular ejection fraction (LVEF) and heart failure requiring monitoring [22,23]. PARP inhibitors are often better tolerated than chemotherapy alone but combining them with chemotherapy increases discontinuation risk and raises anaemia incidence and gastrointestinal toxicity [24,25].

## 4. Potential Mechanisms

### 4.1. Impact of Surgery

In the early postoperative period, patients commonly experience reduced caloric intake, a catabolic surgical stress response, transient hypoxemia driven by residual anaesthetic effects and impaired ventilation, and a systemic inflammatory response characterised by elevated circulating cytokines. The severity of these responses is generally proportional to the extent of surgical trauma and can be reduced by using minimally invasive techniques [26]. These acute alterations may influence drug disposition by modifying plasma protein binding, volume of distribution, hepatic cytochrome P450 activity, and renal clearance, but many components of the acute surgical response are expected to attenuate substantially within the first 1–3 weeks after surgery [27]. This timeframe may precede the initiation of adjuvant chemotherapy, although it should not be assumed that all postoperative consequences have resolved by that time. Indeed, chronic or persistent postoperative sequelae may remain clinically relevant during the adjuvant period. These may include altered gastrointestinal transit and absorptive capacity, persistent catabolism, weight loss, malnutrition, sarcopenia, hypoalbuminemia and changes in hepatic or renal function. However, the extent and duration of these effects are highly heterogeneous and depend on the type and extent of resection, postoperative complications, the patient’s nutritional and functional reserve, and the administered drug [27].

Chronic postoperative sequelae alone are therefore unlikely to fully account for the observed differences in chemotherapy tolerability across surgical procedures with markedly different physiological consequences. Total gastrectomy, for example, may substantially affect gastrointestinal physiology, nutritional status, oral intake, and the absorption of orally administered drugs, whereas breast-conserving surgery generally has a considerably smaller direct impact on gastrointestinal function and systemic metabolism. Rectal resection, which has a comparatively limited impact on oral drug absorption, does not produce noticeably smaller neoadjuvant-to-adjuvant tolerability differences than esophagectomy. The occurrence of a similar neoadjuvant-to-adjuvant tolerability pattern across settings with substantially different surgical burdens suggests that factors beyond the magnitude of anatomical disruption may also contribute. This comparison should not be interpreted as minimising the clinical importance of surgical morbidity; rather, it indicates that postoperative morbidity and pharmacokinetic mechanisms may represent interacting but partially distinct explanations.

Although limited, a few studies have directly compared chemotherapy pharmacokinetics before and after surgery. Specifically, in a dedicated S-1 (viz., oral 5-FU anticancer agent consisting of tegafur, gimeracil, and oteracil) pharmacokinetic study, patients were sampled before and approximately two weeks after gastrectomy. Postoperative AUCs of 5-FU and its dihydropyrimidine dehydrogenase (DPD) inhibitor gimeracil were overall similar to pre-operative values; however, tmax was shorter, and some parameters differed between partial and total gastrectomy [28]. These findings indicate that, once early postoperative instability has subsided, systemic exposure to an oral 5-FU anticancer agent, at a given dose, can be at least as high after gastrectomy as before, despite altered gastrointestinal anatomy. Similarly, other studies reported that gastrectomy does not affect the plasma pharmacokinetics of 5-FU after postoperative S-1 administration [29], or that the observed changes are unlikely to have clinical implications [30].

In contrast, another study reported that when S-1 was administered as adjuvant chemotherapy after total gastrectomy, higher plasma Cmax and AUC were attained; therefore, adverse events must be monitored carefully [31]. It is worth noting that these studies have several caveats: they are limited to an oral fluoropyrimidine regimen, have small sample sizes, involve altered gastrointestinal anatomy, and report inconsistent findings. However, it remains unclear whether these pharmacokinetic alterations result from surgery-related factors (e.g., changes in absorption, gastrointestinal transit, body composition, nutrition, renal function, or hepatic function) rather than the removal of a tumour compartment.

### 4.2. The Tumour as a Drug Sequestration Site

The aberrant vasculature and physiological heterogeneity of the tumour microenvironment can lead to markedly different drug PK than that observed in the systemic circulation [32]. Therefore, tumours may act as a PK compartment or “sink” that removes more drug from the systemic circulation than expected based on the proportion of tissue removed. Conversely, surgery may eliminate the “tumour-sink effect”, potentially altering systemic exposure and toxicity of the drug, even if dosage remains unchanged [33]. This is especially relevant for drugs that undergo significant tumour binding and tumour metabolism [34]. Importantly, tumour drug accumulation should not be equated with a clinically meaningful systemic sink effect. A high tumour-to-plasma concentration ratio may reflect local binding, intracellular trapping, or slow release without materially changing the amount of drug circulating systemically. Most importantly, demonstrating a systemic sink effect would require evidence that tumour burden measurably alters plasma exposure, unbound exposure, clearance, or normal-tissue toxicity, and that these parameters change after tumour removal.

#### 4.2.1. Evidence from Target-Mediated and Radiopharmaceutical Systems: Biological Analogy Rather than Direct Evidence for Cytotoxic Chemotherapy

The concept of a sink effect, called “tumour-sink effect”, was first described in radiotheranostic studies which demonstrated that tumours may indeed behave as PK “sinks,” competing actively with normal tissues for drug uptake [35,36,37]. In metastatic castration-resistant prostate cancer treated with prostate-specific membrane antigen (PSMA)-targeted radioligands, patients with a very high PSMA-positive tumour burden exhibited reduced tracer uptake in normal organs such as the salivary glands, kidneys and spleen. Conversely, as the tumour burden decreased or responded to therapy, uptake in normal organs increased [37]. Similar observations have been reported in melanoma, where a high-affinity melanin drug radioligand, [^131^I]ICF01012, demonstrated that when tumour volume is large, there is a high accumulation of drug in pigmented lesions, with lower activity in non-target organs. As the tumour burden decreases, a relatively larger amount of the radioligand becomes available for normal tissues [38].

Analogously, a “tumour-sink effect” can also be observed with fludeoxyglucose (FDG), a radiotracer used in the medical imaging modality of positron emission tomography (PET). FDG metabolites accumulate preferentially in the tumour [14], demonstrating the concept of a “tumour-sink effect” due to metabolic trapping [34].

Mathematical and systems pharmacology models integrate these observations by explicitly representing tumour and host tissues as distinct, interacting compartments [39]. A systems-based approach to tumour PK has shown that factors such as tumour vascularity, permeability, interstitial pressure and binding capacity significantly affect the distribution of a systemic dose between the tumour and surrounding plasma and normal tissues over time. Computational simulations of tumour growth and treatment response further demonstrate that changes in tumour volume and architecture (for example, after debulking surgery) alter drug distribution patterns and can increase the relative exposure of non-tumour compartments while maintaining the same dosing schedule [39]. These findings demonstrate that tumour burden can influence tracer distribution in selected radiopharmaceutical systems. They, therefore, provide a biological analogy for tumour-associated disposition, but not direct evidence that resection of a conventional solid tumour produces a clinically meaningful change in systemic exposure to cytotoxic chemotherapy. Thus, the relevance of these observations to standard cytotoxic agents remains indirect and requires drug-specific validation.

Beyond radiopharmaceuticals, the “tumour-sink effect” has also been documented for biological anticancer drugs, in which tumour-antigen binding and internalisation create a target-mediated sink that accelerates clearance and lowers systemic exposure as tumour burden increases [40].

This mechanism involves co-internalisation of biological drugs with the target-tumour protein [41], a process favoured by the higher fenestration of tumour capillaries [42]. Furthermore, biological drugs such as monoclonal antibodies have the potential to reach tumours and become sequestered once blood vessel permeability and surface area in the tumour for extravasation are sufficient, which is often achieved through the enhanced permeability and retention (EPR) effect [34].

#### 4.2.2. The Tumour as a Drug Sequestration Site of Chemotherapeutic Drugs

The possibility that the “tumour-sink effect” may also apply to chemotherapeutic drugs must be considered. For instance, it is known that temoporfin, an anticancer photodynamic agent, accumulates in the mitochondria of cancer cells [43]. Similarly, rhodacyanine, a dye with anticancer activity, as well as several other analogues, has been shown to be taken up by cancer cells up to 65 times more efficiently than by nonneoplastic cells [44,45]. Additional examples of chemotherapeutic drugs and the mechanisms that may contribute to the “tumour-sink effect” in solid tumours are summarised in Table 2.

For the analysis of the putative contribution of the “tumour-sink effect” to the pharmacology of the drugs included in the regimens used in gastrointestinal and breast cancer in which alterations in tolerability to perioperative chemotherapy are reported, a more detailed description of the “tumour-sink effect” of fluoropyrimidines, platinum drugs, taxanes and anthracyclines is presented.

To avoid conflating distinct pharmacological observations, we distinguish three levels of evidence throughout this section. Level 1 refers to tumour penetration, local accumulation, intracellular retention, or tumour-selective activation. Level 2 refers to evidence that tumour burden is associated with measurable changes in systemic pharmacokinetics, such as plasma or unbound drug exposure, clearance, or distribution. Level 3 refers to direct evidence that tumour resection changes systemic drug exposure and that this change is associated with normal-tissue toxicity, treatment delivery, or another clinically relevant outcome.

It is noteworthy that the mechanisms described in Table 2, e.g., tumour-selective activation, DNA binding, receptor-mediated internalisation and lysosomal sequestration, should not be treated as interchangeable mechanisms. These mechanisms may contribute to local drug exposure or retention but do not, by themselves, establish a systemic “tumour-sink effect”.

##### Fluoropyrimidine Drugs

Fluoropyrimidines have been fundamental to cancer chemotherapy for over six decades, with 5-FU serving as the prototypical agent for treating colorectal, breast, gastric, and head and neck cancers. Fluoropyrimidines were developed in the 1950s following the observation that rat hepatomas used uracil more rapidly than normal tissues; thus, interference with uracil pathways was seen as a chemotherapy possibility [60]. Fluoropyrimidines are antimetabolite drugs whose active metabolites may be preferentially generated, retained, or incorporated within tumour tissue. These processes provide Level 1 evidence of tumour-associated drug accumulation or local disposition. However, they do not demonstrate that tumour burden modifies systemic pharmacokinetics or that tumour removal increases plasma exposure or toxicity. The most compelling evidence for the existence of a “tumour-sink effect” of fluoropyrimidines comes from studies using ^19^F nuclear magnetic resonance spectroscopy (^19^F-NMR) that directly measure 5-FU concentrations in tumours in vivo. These studies showed that free 5-FU was detectable in most human tumours and in all rabbit tumours, with tumour half-lives of 0.4–2.1 h in human tumours and 1.05–1.22 h in rabbit tumours, significantly longer than the blood half-life of 5–15 min. Critically, no catabolic metabolites were detected in tumours by ^19^F-NMR, contrasting sharply with extensive hepatic catabolism, suggesting that tumours trap free 5-FU without significant local degradation [61] and that a long-lived tumour pool of 5-FU was detected in 6 of 11 tumours, with a half-life of this “trapped” 5-FU ranging from 0.33 to 1.3 h (20 to 78 min), again substantially exceeding the blood half-life [62].

Quantitative measurements of tumour-to-plasma drug concentration ratios provide direct evidence for preferential tumour accumulation of fluoropyrimidines, particularly when 5-FU prodrugs are administered. In patients with advanced breast cancer receiving capecitabine, the tissue distribution factor of 5-FU in tumour was about 1.6-fold greater than in normal tissue [63,64], whereas in patients with primary colorectal tumours, the concentration of 5-FU was on average 3.2 times higher than in adjacent healthy tissue [55].

The capacity of tumours to “sink” 5-FU seems to determine the sensitivity of the tumour to fluoropyrimidines. In patients with liver metastases from colorectal adenocarcinoma, only metastases that could trap 5-FU were more sensitive to 5-FU [65]. These observations support preferential tumour accumulation or retention of fluoropyrimidines in selected experimental and clinical settings. However, they do not establish a tumour-burden-dependent systemic pharmacokinetic effect or demonstrate that tumour resection increases plasma or unbound 5-FU exposure. Direct evidence linking tumour removal to increased systemic exposure, normal-tissue toxicity, or treatment delivery is currently lacking. More alarmingly, the increased impact on non-tumour cells may be greater than that resulting from redistribution of 5-FU retained in the tumour.

Plasma 5-FU is metabolised primarily by dihydropyrimidine dehydrogenase (DPD), encoded by the *DPYD* gene expressed across a wide range of human tissues, but its highest concentrations and activity are found in the liver and mononuclear blood cells. DPD serves as the initial, rate-limiting enzyme in the catabolism of 5-FU, allowing for detoxification and subsequent elimination of 5-FU from the organism. Experimental studies have suggested that changes in 5-FU exposure may influence DPD activity or expression [66,67,68]; however, the clinical relevance of this relationship in the postoperative setting remains uncertain. There is no direct human evidence demonstrating that tumour resection causes DPD downregulation that subsequently increases systemic 5-FU exposure or toxicity. This pathway should therefore be regarded as speculative and should not be interpreted as evidence of a post-resection “tumour-sink effect”.

##### Platinum (Pt)-Based Anticancer Drugs

Platinum (Pt)-based anticancer agents remain a cornerstone of contemporary chemotherapy and are frequently incorporated into treatment regimens where tolerability may differ between neoadjuvant and adjuvant settings. Growing evidence indicates that cancer cells can concentrate Pt-based drugs to levels substantially higher than those observed in surrounding normal tissues. This preferential accumulation arises from a combination of passive diffusion and active transport processes and provides evidence of local platinum disposition within tumour cells and the tumour microenvironment; whether this accumulation is sufficient to produce a PK “tumour-sink effect” with potential clinical relevance after tumour resection remains unknown.

The intracellular accumulation of platinum compounds is mediated by multiple transport systems [69]. Among these, the copper transporter CTR1 (SLC31A1) plays a central role in facilitating the uptake of clinically used platinum agents at physiologically relevant concentrations [70,71]. Increased CTR1 expression has been associated with enhanced platinum accumulation in tumour cells and improved therapeutic response in patients, as demonstrated in ovarian carcinoma models. In parallel, organic cation transporters (OCTs) significantly contribute to platinum uptake; for example, OCT1 and OCT2 expression has been shown to increase oxaliplatin accumulation by 5.6-fold and 23.9-fold, respectively, relative to control cells [72].

Importantly, platinum sequestration is not restricted to malignant cells. Cancer-associated fibroblasts have also been shown to accumulate platinum compounds, contributing to a broader tumour-level reservoir [73]. This observation supports the concept that the entire tumour microenvironment may function as a PK compartment for Pt-based drugs. The resection of a platinum-accumulating tumour mass provides experimental evidence for the “tumour-sink effect”, as removal of the tumour reservoir could theoretically alter systemic drug distribution, potentially leading to increased exposure of non-tumour tissues and a heightened risk of toxicity and decreased tolerability. From a “tumour-sink” perspective, when macroscopic disease is still present (neoadjuvant setting), part of each cisplatin or oxaliplatin dose is diverted into and retained by tumour tissue, whereas after complete resection this compartment disappears, and a larger fraction of the same dose is consequently distributed, metabolised, and cleared by normal organs alone. This shift might increase systemic exposure (and, therefore, toxicity) for any given “standard” dose in the immediate adjuvant period. However, the accumulation of platinum compounds within tumour cells and stromal components provides Level 1 evidence of local drug retention. It does not, however, constitute direct evidence that resection of the tumour changes systemic platinum exposure, clearance, or normal-tissue toxicity.

##### Taxanes

Taxanes, including paclitaxel and docetaxel, are widely used chemotherapeutic agents with established efficacy in multiple cancer types. Mechanistically, taxanes interfere with microtubule function by promoting tubulin polymerisation, and stabilizing microtubules inducing mitotic arrest and inhibiting cell proliferation [74]. The antitumour activity of taxanes extends beyond their antimitotic properties because microtubules integrate the cytoskeleton and influence polarity, morphology, epithelial-to-mesenchymal transition (EMT), oncogenic signalling, and intracellular trafficking [75].

Despite their structural similarities, paclitaxel and docetaxel exhibit particularities in their pharmacodynamic and PK profiles [76]. One of the most fundamental pharmacodynamic differences between paclitaxel and docetaxel lies in their affinity for the β-tubulin binding site. Multiple studies have demonstrated that docetaxel exhibits approximately 2-fold greater binding affinity to β-tubulin compared to paclitaxel [77]. This superior tubulin binding capacity contributes to docetaxel’s enhanced cytotoxic potency and may explain some of its clinical advantages in certain tumour types [76,78].

Paclitaxel is characterised by a very large, dose-dependent apparent volume of distribution and non-linear PK, with small dose increments leading to disproportionate increases in plasma Cmax (maximum plasma concentration) and AUC (area under the curve).

In vitro studies using tumour cell models have demonstrated extensive intracellular accumulation of paclitaxel, with reported cellular levels reaching 50- to 1000-fold higher than the initial extracellular drug concentrations [49,50] (see Table 2). Complementary clinical PK studies in patients with gynaecological malignancies, including ovarian, endometrial, and cervical cancers, have shown that paclitaxel is preferentially retained within tumour tissues, with lower or no accumulation observed in the corresponding normal tissues, supporting its effective tumour exposure in vivo [79]. A biodistribution study with nanoparticle formulations of paclitaxel also showed differential retention and efflux between tumour and non-tumour tissues, with multidrug-resistant tumours exhibiting altered uptake/clearance patterns compared with sensitive lesions [38,80]. These studies emphasise that tumour tissue acts as a major PK and pharmacodynamic compartment, capable of binding and slowly releasing paclitaxel over time [80].

Docetaxel also demonstrates particularities in its PK. Preclinical studies have consistently shown that docetaxel exhibits greater cellular uptake, slower efflux from tumour cells, and consequently longer intracellular retention time [76,78,81]. Following rapid tissue uptake, the elimination of docetaxel in normal tissues was found to be biphasic with terminal half-lives ranging from 2.2 to 4.5 h, whereas the elimination half-life of docetaxel from tumour sites is approximately 22 h, suggesting long tumour site retention [82]. The mechanisms underlying prolonged tumour retention are not fully elucidated, but the intracellular binding to tubulin, effectively sequestering drug within cells, may be a major contributor. This extended retention suggests that tumour tissue may act as a pharmacological reservoir, maintaining local drug concentrations even as systemic levels decline, and may constitute a mode of existence of the “tumour-sink effect” of taxanes (particularly docetaxel because of its higher affinity) in sensitive tumours. In addition to the prolonged tumour retention, the finding that tumour exposures increase proportionately with dose suggests that tumour tissue has substantial capacity to accumulate drug without saturation at clinically relevant doses [83].

##### Anthracyclines

Anthracyclines are effective treatments against multiple solid tumours, such as breast, ovarian and GI cancers. Regarding their mechanisms of action, anthracyclines exert pleiotropic effects, including DNA intercalation and chromatin damage, topoisomerase II poisoning, redox-mediated mechanisms, alterations in calcium homeostasis, generation of reactive oxygen species, and mitochondrial dysfunction (reviewed in [84]). As mentioned above (see Table 2), DNA intercalation and lysosomal sequestration may contribute to higher intracellular doxorubicin accumulation in tumour cells. In patients with locally advanced breast cancer, intratumoral doxorubicin concentrations measured 48 h after administration were approximately 50–300-fold higher than plasma concentrations, indicating prolonged retention in tumour tissue [85].

## 5. Impact of Surgery-Induced Pharmacological Changes on the Tolerability of Anticancer Drugs

Based on what has been presented above, surgery is expected to influence the tolerability of systemic anticancer therapies not only through physiological perturbations but also through alterations in drug disposition (by eliminating the “tumour-sink effect”).

From a pharmacokinetic perspective, the “tumour-sink effect” could influence drug disposition by acting as a peripheral distribution compartment, but whether this produces a measurable change in systemic exposure remains unknown. Although the relative mass of the tumour is typically small compared with total body weight, this argument alone is insufficient to dismiss clinical relevance, as drug distribution is governed not only by mass but also by tissue-specific affinity, vascular permeability, transporter expression, and binding capacity. However, detection of high drug concentrations within tumour tissue demonstrates local sequestration or retention, but it does not, by itself, establish that tumour removal will increase plasma concentrations, systemic AUC, or toxicity in normal tissues. The magnitude of any systemic effect cannot currently be quantified because the relevant parameters—including tumour mass, tumour-to-plasma concentration ratios, unbound drug fractions, intracellular retention, effective distribution volume, timing of resection, and drug-specific clearance—are not sufficiently characterised in the perioperative setting. Consequently, the available evidence does not allow reliable estimation of how tumour removal affects plasma Cmax, AUC, clearance, or normal-tissue toxicity for conventional cytotoxic chemotherapy.

The critical issue is whether eliminating this compartment results in a measurable increase in systemic exposure (e.g., AUC or peak plasma concentrations) sufficient to shift patients along the steep portion of the exposure–toxicity relationship. A clinically important change in Cmax or AUC would require additional conditions, such as high-affinity or irreversible binding, sustained release from tumour or stromal compartments, target-mediated disposition, non-linear elimination, saturation of clearance pathways, or a substantial effect on the distribution or clearance of the administered drug. For many cytotoxic and targeted agents, therapeutic windows are narrow and used doses approach toxicity thresholds. Under these conditions, even modest increases in systemic exposure may translate into a clinically meaningful increase in adverse events.

This consideration becomes more relevant in combination chemotherapy, where multiple agents with overlapping toxicities are administered simultaneously. If the tumour contributes to the distribution or sequestration of several drugs within a regimen, its removal may lead to a parallel increase in systemic exposure across agents. Given the non-linear and often supra-additive nature of toxicity in combination regimens, this could result in a disproportionate escalation in adverse effects, rather than a simple additive increase.

Notably, clinical contexts in which perioperative tolerability is most compromised often involve regimens composed of drugs with high tumour uptake or distribution sensitivity, as described for protocols such as FLOT. This observation is consistent with the hypothesis that loss of the “tumour-sink effect” may contribute to increased systemic exposure and overlapping toxicities. While direct quantitative evidence remains limited, the mechanistic plausibility and pharmacological context support the need for careful dose optimisation and monitoring in the postoperative setting.

## 6. Strategies to Increase Tolerability to Adjuvant Chemotherapy

Strategies to mitigate the decline in tolerability from neoadjuvant to adjuvant chemotherapy should not disregard the possibility that the tumour materially contributes to the PK of the administered drugs. Importantly, these are potential strategies rather than validated interventions specifically targeting a “tumour-sink mechanism” which remains a mechanistic hypothesis requiring clinical validation.

Addressing reduced tolerability in the perioperative setting requires a shift away from a one-size-fits-all paradigm toward approaches that identify the agents most affected by tumour removal and support dose adjustment based on therapeutic drug monitoring (TDM) and the severity of adverse drug reactions, thereby preserving oncologic benefit. Treatment de-escalation is central to this strategy because it reduces excess toxicity, prolongs recovery, and limits avoidable treatment burden when lower-intensity regimens are likely to be sufficient. In parallel, biologically informed personalisation integrates tumour- and patient-specific characteristics to optimise treatment intensity, sequence, and duration according to the expected balance of benefit and harm.

### 6.1. De-Escalation

De-escalation in oncology refers to the deliberate reduction in treatment intensity, duration, frequency, or extent in patients for whom full-intensity therapy is unlikely to provide additional meaningful benefit. In modern practice, de-escalation is increasingly linked to biological selection, allowing patients with favourable clinicopathologic or molecular features to avoid overtreatment without compromising outcomes [86,87].

There are several examples in the literature showing that de-escalation can be feasible and clinically meaningful. In early HER2-positive breast cancer, the PHERGain strategy tested whether some patients could avoid chemotherapy by using response-adapted therapy and achieved a de-escalated approach in a subset of patients without obvious compromise in short-term outcomes [88]. In metastatic breast cancer, the X-7/7 trial showed that a modified capecitabine schedule reduced toxicity without worsening survival endpoints [89]. This de-escalation paradigm is also emerging in gastrointestinal malignancies [87]. In gastric cancer, the AMC0201 randomised phase III trial showed that intensifying adjuvant chemotherapy after D2 gastrectomy was safe but did not improve recurrence-free or overall survival, supporting the rationale for less intensive approaches in appropriately selected patients [90].

Together, these data indicate that adjuvant de-escalation is no longer merely theoretical; rather, it is becoming a clinically and methodologically grounded strategy to align efficacy with tolerability and long-term patient outcomes.

### 6.2. Biologically and Therapeutically Informed Personalisation

Biologically and therapeutically informed personalisation is increasingly replacing the traditional “one-size-fits-all” approach to adjuvant chemotherapy, particularly in fluoropyrimidine-based regimens.

In CRC, tumour-specific biomarkers such as *TYMS* expression or copy-number changes have been associated with differential benefit from 5-FU-based adjuvant therapy, suggesting that some patients derive meaningful benefit while others may be exposed to toxicity without clear gain [91,92]. More broadly, molecular biomarkers can help identify patients most likely to benefit from treatment, allowing postoperative therapy to be matched more closely to recurrence risk and tumour biology rather than applied uniformly [91,92,93,94].

TDM provides a complementary, pharmacokinetically driven approach to personalisation, especially for 5-FU, where interpatient variability in exposure is large and conventional body-surface-area dosing is imprecise. Reviews and clinical data show that 5-FU TDM is feasible and can reduce grade 3/4 toxicity while improving the likelihood of achieving an exposure range associated with efficacy. In elderly gastrointestinal cancer patients, exposure-guided dose adjustment has also been shown to increase the proportion of patients within the therapeutic range from the first to subsequent cycles, supporting the practical value of PK-guided dosing in routine care [95,96,97].

The clinical rationale for combining biomarker guidance with TDM is strong: biomarkers address which patients should receive adjuvant therapy, whereas TDM addresses how much therapy should be given to patients. This distinction is important because tumour biology and host pharmacology contribute differently to treatment benefit and toxicity, and both must be considered to preserve efficacy while minimising harm. In this framework, biomarkers such as *TYMS* and *DPYD*-related markers, together with TDM, create a more rational basis for postoperative treatment adaptation than empirical dose reduction alone [91,95,97,98].

Taken together, evidence supports a precision-adjuvant model in which molecular profiling and TDM are used to individualise drug choice and dose intensity [91,95,97,99]. Although the strongest data are available for fluoropyrimidines, the underlying principle is likely transferable to other adjuvant regimens as biomarker validation and exposure-guided strategies continue to mature. This biologically and therapeutically informed approach is particularly attractive in the adjuvant setting, where the therapeutic window is narrow, and the cost of overtreatment may be substantial in terms of toxicity, quality of life, and treatment adherence.

## 7. Limitations and Future Perspectives

Despite the well-recognised differences in tolerability between neoadjuvant and adjuvant chemotherapy, the biological and clinical mechanisms responsible for this disparity remain incompletely understood. Postoperative complications, infections, delayed recovery, malnutrition, sarcopenia, reduced performance status, organ dysfunction, and early recurrence may substantially impair the ability to initiate or complete adjuvant treatment and should not be regarded as secondary considerations. Surgery-related factors alone cannot adequately explain the consistent pattern observed across gastrointestinal malignancies and other tumour types. These factors may also interact with persistent changes in gastrointestinal function, protein binding, drug distribution, metabolism, and clearance.

This review presents limitations that should be considered when interpreting its findings. First, this is a narrative, non-systematic review rather than a systematic review or meta-analysis; therefore, the evidence has been qualitatively synthesised, and selection bias in identifying and interpreting the available literature cannot be ruled out. Second, the clinical evidence is heterogeneous regarding cancer types, disease stages, chemotherapy regimens and sequences, surgical procedures, patient characteristics, and clinical endpoints, limiting cross-study comparability and preventing a uniform estimate of differences in tolerability between neoadjuvant and adjuvant treatment. This is particularly relevant for gastrointestinal and breast cancers, given the substantially different extent and physiological consequences of surgery.

Clinical comparisons, particularly from retrospective or observational cohorts, are also susceptible to confounding by indication and selection bias. Patients receiving postoperative chemotherapy may differ from those who do not, as treatment initiation or completion can be influenced by postoperative complications, performance and nutritional status, comorbidities, disease progression, patient preferences, and clinical decision-making. Therefore, postoperative non-completion cannot be attributed to chemotherapy toxicity alone, as postoperative deterioration, complications, impaired oral intake, and changes in performance status may also contribute to treatment modification or omission.

The mechanistic interpretation of tumour resection is further limited by the scarcity of direct clinical evidence that removal of the primary tumour increases systemic exposure to conventional cytotoxic drugs and, consequently, normal-tissue toxicity. Direct perioperative pharmacokinetic comparisons also remain limited. As such, the proposed relationship between tumour removal, redistribution of drug exposure, and postoperative toxicity should be regarded as biologically plausible rather than an established causal mechanism. Evidence supporting a “tumour-sink effect” derives partly from radioligand and FDG studies, antibody-based therapies, mathematical models, and preclinical experiments. Although these provide proof of concept that tumour burden can influence drug or tracer distribution, extrapolation to conventional cytotoxic chemotherapy is limited by differences in molecular targets, transport, tissue binding, metabolism, and pharmacokinetics. Evidence from fluoropyrimidines, platinum compounds, taxanes, and anthracyclines is more directly relevant, but does not establish that tumour removal consistently increases systemic exposure or normal-tissue toxicity after surgery.

Finally, tumour resection does not necessarily eliminate all malignant tissue; residual micrometastatic disease or persistent tumour-associated compartments may continue to influence drug distribution, metabolism, or pharmacodynamics. Consequently, changes in tumour-to-normal-tissue exposure cannot be attributed solely to the removal of the macroscopic primary tumour. Prospective studies integrating perioperative pharmacokinetic sampling with quantitative assessment of tumour burden, residual disease, tissue drug concentrations, and toxicity are needed to determine whether tumour resection produces clinically meaningful changes in cytotoxic-drug exposure and to clarify its contribution relative to postoperative physiological vulnerability.

In this work, we do not intend to replace these established clinical explanations with a single pharmacokinetic mechanism. Rather, we present the ‘’tumour-sink effect’’ and related changes in drug disposition as additional, biologically plausible hypotheses that may complement postoperative morbidity and help explain interindividual and inter-surgical variability in chemotherapy tolerability. 

The available evidence does not yet establish that tumour removal increases systemic exposure to or toxicity of conventional cytotoxic agents in the adjuvant setting. Postoperative treatment delivery is frequently compromised, particularly after major gastrointestinal cancer surgery, but this appears to reflect multiple interacting factors, including surgical complications, postoperative physiological and functional deterioration, nutritional status, sarcopenia, organ function, comorbidities, disease progression, and treatment-related toxicity. The “tumour-sink hypothesis” provides a biologically plausible explanation for how changes in tumour burden might influence drug disposition, whereby removal of tumour-associated sequestration sites could alter the fraction of drug available for distribution to normal tissues. However, direct clinical evidence supporting this mechanism for conventional cytotoxic agents remains limited, and it should therefore be regarded as a hypothesis requiring prospective validation rather than as an established explanation for the reduced tolerability of adjuvant compared with neoadjuvant chemotherapy.

Future studies should prospectively evaluate patients before and after curative tumour resection using paired pharmacokinetic and pharmacodynamic assessments. Ideally, these studies should integrate treatment timing and surgical characteristics with quantitative measures of tumour burden and residual disease, nutritional status, sarcopenia, organ function, albumin concentration, inflammatory markers, pharmacogenetic characteristics, total and unbound drug exposure, toxicity, and delivered dose intensity. Such an integrated approach would help determine whether tumour resection produces clinically meaningful changes in cytotoxic-drug disposition and whether these changes contribute independently to postoperative toxicity or treatment modification.

Personalised treatment strategies, including biomarker-informed selection, dose optimisation, and therapeutic drug monitoring, may ultimately help improve the balance between efficacy and toxicity. Genomic and molecular profiling may help identify patients less likely to benefit from standard-intensity adjuvant therapy, while TDM may help optimise drug exposure where clinically appropriate. Dose de-escalation could also be investigated as a means of reducing unnecessary toxicity and treatment burden while preserving efficacy. However, these approaches should not be implemented specifically based on the “tumour-sink hypothesis”, and whether they can compensate for tumour-removal-associated changes in drug disposition remains to be established.

Figure 1 summarises the multiple mechanisms that may contribute to the reduced tolerability of adjuvant compared with neoadjuvant chemotherapy, including the proposed “tumour-sink hypothesis”, alongside potential strategies for investigation. Ultimately, prospective adjuvant trials should determine whether personalised approaches can improve treatment tolerability and long-term outcomes without compromising oncological efficacy. Progress in this area will require close collaboration between basic scientists and clinicians to integrate mechanistic, pharmacokinetic, pharmacodynamic, and clinical evidence and establish whether tumour-associated drug sequestration has a clinically meaningful role in postoperative chemotherapy.

## 8. Conclusions

Surgery-related factors alone are unlikely to account for the consistent differences in chemotherapy tolerability observed between neoadjuvant and adjuvant treatment across gastrointestinal malignancies and other tumour types. The proposed “tumour-sink effect,” and the resulting changes in drug disposition following tumour resection, may provide an additional explanation: removal of tumour-associated drug-sequestration sites could increase systemic exposure of normal tissues to cytotoxic agents and thereby exacerbate postoperative morbidity. The clinical relevance and magnitude of this mechanism warrant further investigation.

## Figures and Tables

**Figure 1:**
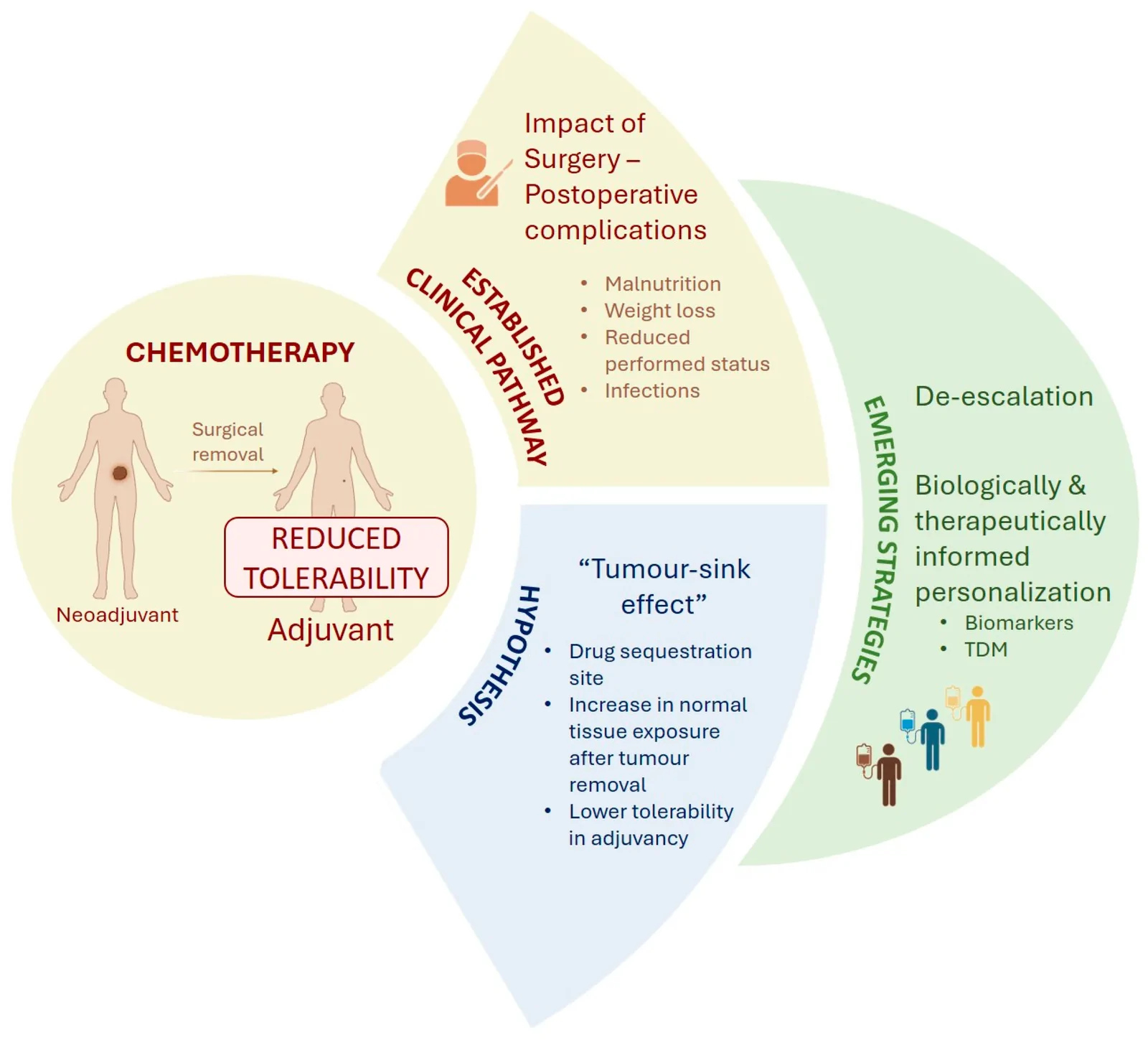
Schematic representation of the proposed mechanisms underlying reduced adjuvant chemotherapy tolerability. Postoperative morbidity, nutritional impairment, organ dysfunction, and delayed recovery may reduce treatment tolerance. In addition, tumour resection may alter pharmacokinetics, tissue distribution, and drug–host interactions, potentially narrowing the therapeutic index. Emerging strategies to address these issues include de-escalation and biologically and therapeutically informed personalisation.

**Table 1:** Summary of clinical studies comparing neoadjuvant and adjuvant chemotherapy.

Cancer Type	Design, Setting, Sample and Population	Regimen and Planned Cycles	Postoperative Initiation	Postoperative Completion	Relative Dose Intensity/Treatment Modification	Grade 3–4 Adverse Events and Toxicity-Related Discontinuation	Non-Toxicity Related Non-Delivery and Postoperative Complications	Main Limitations and Risk Confounding	Refs.
Gastric	Retrospective cohort; n = 171; locally advanced, resectable gastric cancer	FLOT: 4 preoperative + 4 postoperative cycles	Not reported separately	105/171 (61.4%) completed all planned postoperative cycles	37 patients switched to FOLFOX; relative dose intensity not reported	Haematological and neurological toxicity were among the reported reasons for regimen modification; toxicity-specific discontinuation not separately quantified	29/171 received no postoperative chemotherapy, mainly because of postoperative deterioration and complications; poor ECOG status and impaired oral intake also contributed	Retrospective design; non-randomised treatment delivery; confounding by postoperative fitness, comorbidity, and disease severity	[2]
	Propensity-matched comparison; locally advanced, resectable gastric cancer; analysis population n = 37 per group	Standard perioperative FLOT 4 + 4 versus total neoadjuvant FLOT × 8	In the FLOT 4 + 4 group, 30/37 (81.1%) initiated postoperative treatment; 7/37 (18.9%) did not	25/37 (67.6%) completed all planned cycles in the FLOT 4 + 4 group versus 33/37 (89.1%) in the total neoadjuvant group	Relative dose intensity not reported; all-cycle completion reported	Intolerance reported, but toxicity-specific discontinuation not fully separated	Postoperative complications and inability to initiate treatment were reported	Propensity-matched observational comparison; small analysis population; different treatment sequencing	[3]
	Real-world cohort; n = 343; resectable gastric cancer	Modified FLOT: 4 preoperative + 4 postoperative cycles	Not reported separately	264/343 (77.0%) completed planned postoperative chemotherapy	Dose reductions, regimen changes, and discontinuations reported; relative dose intensity not reported	Grade 3–4 diarrhoea and febrile neutropenia reported; toxicity-specific discontinuation not fully separated	Postoperative frailty and other clinical factors contributed to non-delivery	Observational study; incomplete separation of toxicity-related and non-toxicity-related treatment changes	[4]
	Phase II perioperative study; resectable gastric adenocarcinoma	Nab-paclitaxel plus FOLFOX: 6 preoperative cycles followed by postoperative treatment	Exact denominator should be reported from the source study	22/44 (50.0%) resumed the same postoperative regimen; approximately one-third received no postoperative chemotherapy	Regimen changes reported; relative dose intensity not reported	Tolerance issues reported; exact toxicity-related discontinuation should be specified if available	Performance-status decline and insufficient response also contributed	Phase II study; small sample; postoperative analysis population may differ from enrolled population	[5]
	Randomised phase III trial; resectable gastro-oesophageal cancer	ECF: 3 preoperative + 3 postoperative cycles	137/250 (54.8%) initiated postoperative chemotherapy, according to the reported denominator	Exact completion denominator and definition should be reported; approximately 40–42% completed all six cycles	Relative dose intensity not reported	Previous toxic effects and postoperative treatment limitations reported	Disease progression, early death, patient choice, postoperative complications, lack of response, and worsening comorbidity	Older regimen and treatment era; completion affected by multiple clinical and disease-related factors	[6]
	Randomised phase II/III trial; locally advanced, resectable disease	FLOT: 4 preoperative + 4 postoperative cycles	Exact number initiating postoperative treatment should be reported if available	162/356 (45.5%) completed all four postoperative cycles; 320/356 (89.9%) completed all four preoperative cycles	Relative dose intensity by phase should be added if reported	Toxicity reported as one reason for non-delivery; toxicity-specific discontinuation should be separated if available	Disease progression, early death, lack of efficacy, patient request, and other postoperative factors	Trial-level comparison; postoperative completion is not equivalent to toxicity or tolerability	[7]
Colorectal	Pooled analysis; n = 4046; postoperative adjuvant chemotherapy	Various fluoropyrimidine- and oxaliplatin-based adjuvant regimens	Not applicable	Not consistently reported	Dose reductions, suspensions, and discontinuations reported	Grade 0–4 toxicity: 18%, 24%, 37%, 19%, and 1.4%, respectively; toxicity-related discontinuation not consistently reported	Reasons for non-delivery should not be inferred from adverse-event grades	Adjuvant-only study; contextual evidence for postoperative toxicity, not direct evidence of neoadjuvant-versus-adjuvant differences	[8]
	Phase II randomised trial; n = 108; locally advanced rectal cancer	CAPOX: postoperative CAPOX after chemoradiation and surgery versus preoperative CAPOX followed by chemoradiation and surgery	Report separately by treatment arm if available	Compliance: 28/37 (75.7%) in the postoperative arm versus 52/54 (96.3%) in the preoperative arm, according to the reported analysis population	Relative dose intensity not reported	Grade 3–4 toxicity: 54% in the postoperative arm versus 19% in the preoperative arm; grade 3 diarrhoea: 16% versus 4%	Multimodality sequencing and postoperative recovery may influence delivery	Small phase II study; chemoradiation and surgery make direct attribution to chemotherapy timing difficult	[9,10]
Breast	Meta-analysis of 14 randomised trials; approximately 5500 women with operable breast cancer	Similar regimens administered preoperatively or postoperatively	Report according to the individual source studies	Report according to the individual source studies	Relative dose intensity not consistently reported	Adverse effects, serious infection, and cardiotoxicity reported; toxicity-related discontinuation not consistently available	Postoperative complications and other causes of non-delivery varied across trials	Historical evidence; heterogeneous regimens, endpoints, and treatment eras; limited applicability to current subtype-specific treatment	[1]
	Randomised phase III trial; n = 1355; operable breast cancer	Doxorubicin–paclitaxel followed by CMF, administered as primary systemic or adjuvant treatment	416/448 (92.9%) in the primary systemic group, according to the reported analysis	798/876 (91.1%) in the adjuvant group, according to the reported analysis	Relative dose intensity should be added if available	Grade 3–4 toxicity below 5% across arms	Non-delivery reasons should be reported separately if available	Historical regimen; not representative of all contemporary HER2-positive or triple-negative treatment strategies	[11]

Note: “Postoperative initiation” indicates that at least one postoperative treatment was administered. “Postoperative completion” indicates completion of all planned postoperative cycles according to the study definition. “Relative dose intensity” refers to the delivered dose relative to the planned dose over time. Grade 3–4 adverse events describe toxicity severity and are not equivalent to treatment discontinuation or non-completion. Percentages should be interpreted within the denominator and analysis population reported for each study and should not be compared directly when endpoint definitions, treatment phases, or populations differ. “Not reported” indicates that the original study did not provide the relevant endpoint or that it was not available in the reviewed report.

**Table 2:** Representative examples of anticancer drugs and biological mechanisms involved in preferential tumour accumulation and their potential contribution to a “tumour-sink effect”.

Drug Class & Representative Drugs	Proposed Mechanism Potentially Contributing to a “Tumour-Sink Effect”	Evidence Type	Level of Evidence	Relevance to Post-Resection Systemic PK	Evidence of Altered Systemic Exposure After Resection	Refs.
*Anthracyclines* - doxorubicin	DNA intercalation and lysosomal sequestration prolong intracellular retention	In vitro and in vivo animal, human tumour-tissue pharmacology	Level 1: local accumulation/retention	Indirect	Absent	[42,46,47,48]
*Taxanes* - paclitaxel, docetaxel	High-affinity binding to microtubules and slow intracellular release increase tumour retention	In vitro, animal, selected human tissue studies Intracellular retention in cancer cells (e.g., paclitaxel accumulates intracellularly 50- to 1000-fold in cancer cells) and heterogeneous tissue penetration	Level 1: local accumulation/retention	Indirect	Absent	[49,50]
*Platinum compounds* - cisplatin, oxaliplatin	Irreversible covalent binding to DNA results in prolonged intracellular retention	In vitro and in vivo animal, tumour-cell and stromal studies Extensive DNA adduct formation demonstrated in tumour tissue	Level 1: local accumulation/retention	Indirect	Absent	[48,51]
*Mitomycin C*	Preferential activation under hypoxic conditions together with irreversible DNA cross-linking	In vitro and in vivo animal studies Evolving hypoxic tumour regions and DNA cross-linking	Level 1: local activation/retention	Indirect and drug-specific	Absent	[52]
*Methotrexate*	Intracellular polyglutamation generates metabolites that remain trapped within tumour cells for prolonged periods	Biochemical, in vitro, and pharmacological studies of intracellular polyglutamation	Level 1: intracellular retention	Indirect and drug-specific	Absent	[53]
*Fluoropyrimidines* - capecitabine (oral prodrug of 5-FU)	Tumour-selective metabolic activation through thymidine phosphorylase (TP) results in higher local concentrations of active metabolites	Human tumour-tissue pharmacology; in vitro and experimental studies; limited clinical PK studies after gastrectomy	Level 1: local accumulation/retention; Level 2 limited for surgery-associated PK	Direct for selected tissue-distribution observations; indirect for a tumour-sink mechanism	Limited and inconsistent after gastrectomy; absent for tumour-resection-associated systemic exposure in conventional cytotoxic therapy	[54,55]
*Tyrosine kinase inhibitors* - sunitinib	Lysosomal sequestration of weakly basic chemotherapeutics produces marked intracellular accumulation	In vitro and translational studies of lysosomal sequestration	Level 1: intracellular sequestration	Indirect and not directly transferable to conventional cytotoxic chemotherapy	Absent for post-resection exposure	[56]
*Anthracenediones* - mitoxantrone	Combined lysosomal sequestration and DNA binding prolong intracellular residence time	In vitro Experimental evidence of intracellular accumulation and delayed drug efflux	Level 1: intracellular retention	Indirect and speculative	Absent	[48,56]
*Antibody–drug conjugates* - trastuzumab- emtansine (T-DM1)	HER2-mediated internalisation followed by intracellular release of maytansinoid (DM1) results in selective tumour uptake	Clinical and translational studies of receptor-mediated internalisation and target-mediated disposition	Level 2: in selected biological systems	Indirect analogy; not directly transferable to conventional cytotoxic chemotherapy	Available for selected target-mediated biologic systems; absent for post-resection cytotoxic chemotherapy	[57]
*Nanomedicines* - pegylated liposomaldoxorubicin	EPR effect promotes preferential accumulation within tumour interstitium	In vivo preclinical studies; tumour vascular permeability and enhanced permeability and retention	Level 1: tumour-associated distribution	Indirect and formulation-specific	Absent for post-resection systemic exposure	[58,59]

Note: Level 1 refers to tumour penetration, local accumulation, intracellular retention, or tumour-selective activation. Level 2 refers to evidence that tumour burden is associated with altered systemic pharmacokinetics, such as plasma or unbound exposure, clearance, or distribution. Level 3 would require direct evidence that tumour resection changes systemic exposure and that this change is linked to normal-tissue toxicity, treatment delivery, or another clinically relevant outcome. For the conventional cytotoxic classes considered here, Level 3 evidence is currently unavailable. The evidence categories are descriptive. High local drug concentrations should not be interpreted as proof of a clinically meaningful systemic “tumour-sink effect”.

## Data Availability

No new data were created or analysed in this study. Data sharing is not applicable to this article.

## Abbreviations

The following abbreviations are used in this manuscript:

ASCO	American Society of Clinical Oncology
AUC	Area under the curve
CMF	Cyclophosphamide, methotrexate, and 5-fluorouracil
C_max_	Maximum plasma concentration
CRC	Colorectal cancer
CTCAE	Common Terminology Criteria for Adverse Events
CTR1	Copper transporter
ctDNA	Circulating tumour DNA
ECF	Epirubicin, cisplatin, and 5-fluorouracil
ECOG	Eastern Cooperative Oncology Group
ECTO	European Cooperative Trial in Operable Breast Cancer
ECX	Epirubicin, cisplatin, and capecitabine
EMT	Epithelial-to-mesenchymal transition
EPR	Enhanced permeability and retention
ESMO	European Society for Medical Oncology
5-FU	5-Fluorouracil
FDG	Fludeoxyglucose
FLOT	5-Fluorouracil, leucovorin, oxaliplatin, and docetaxel
FOLFOX	5-Fluorouracil, leucovorin, and oxaliplatin
FOXAGAST	5-Fluorouracil, leucovorin, oxaliplatin, and nab-paclitaxel
ICIs	Immune checkpoint inhibitors
OCTs	Organic cation transporters
PET	Positron emission tomography
PK	Pharmacokinetics
PSMA	Prostate-specific membrane antigen
TDM	Therapeutic drug monitoring
TP	Thymidine phosphorylase
